# A new syndrome of moyamoya disease, kidney dysplasia, aminotransferase elevation, and skin disease associated with de novo variants in *RNF213*

**DOI:** 10.1002/ajmg.a.62215

**Published:** 2021-05-07

**Authors:** Alanna Strong, Gina O'Grady, Evelyn Shih, Jonathan R. Bishop, Kathleen Loomes, Tamir Diamond, Erum A. Hartung, William Wong, Sanmati Cuddapah, Anne Marie Cahill, Cuiping Hou, Diana Slater, Courtney Vaccaro, Deborah Watson, Dong Li, Hakon Hakonarson

**Affiliations:** ^1^ Division of Human Genetics Children's Hospital of Philadelphia Philadelphia Pennsylvania USA; ^2^ The Center for Applied Genomics Children's Hospital of Philadelphia Philadelphia Pennsylvania USA; ^3^ Pediatric Neuroservices, Starship Children's Health Auckland District Health Board Auckland New Zealand; ^4^ Division of Neurology, Department of Pediatrics Children's Hospital of Philadelphia Philadelphia Pennsylvania USA; ^5^ Department of Neurology, Perelman School of Medicine University of Pennsylvania Philadelphia Pennsylvania USA; ^6^ Department of Pediatric Gastroenterology, Starship Child Health Auckland District Health Board Auckland New Zealand; ^7^ Division of Gastroenterology, Hepatology, and Nutrition Children's Hospital of Philadelphia Philadelphia Pennsylvania USA; ^8^ Division of Gastroenterology, Hepatology, and Nutrition University of Pennsylvania Philadelphia Pennsylvania USA; ^9^ Division of Nephology, Department of Pediatrics Children's Hospital of Philadelphia Philadelphia Pennsylvania USA; ^10^ Department of Pediatrics, Perelman School of Medicine University of Pennsylvania Philadelphia Pennsylvania USA; ^11^ Department of Pediatric Nephrology, Starship Child Health Auckland District Health Board Auckland New Zealand; ^12^ Division of Interventional Radiology, Department of Radiology Children's Hospital of Philadelphia Philadelphia Pennsylvania USA; ^13^ Department of Radiology, Perelman School of Medicine University of Pennsylvania Philadelphia Pennsylvania USA; ^14^ Division of Pulmonary Medicine Children's Hospital of Philadelphia Philadelphia Pennsylvania USA

**Keywords:** elevated aminotransferases, erythema multiforme, kidney dysplasia, moyamoya disease, RNF213

## Abstract

Ring‐finger protein 213 (*RNF213*) encodes a protein of unknown function believed to play a role in cellular metabolism and angiogenesis. Gene variants are associated with susceptibility to moyamoya disease. Here, we describe two children with moyamoya disease who also demonstrated kidney disease, elevated aminotransferases, and recurrent skin lesions found by exome sequencing to have de novo missense variants in *RNF213*. These cases highlight the ability of *RNF213* to cause Mendelian moyamoya disease in addition to acting as a genetic susceptibility locus. The cases also suggest a new, multi‐organ *RNF213*‐spectrum disease characterized by liver, skin, and kidney pathology in addition to severe moyamoya disease caused by heterozygous, de novo C‐terminal *RNF213* missense variants.

## 1 INTRODUCTION

Moyamoya disease (MMD) is a cerebral vasculopathy characterized by pathological narrowing of the internal carotid arteries with consequent cerebral hypoperfusion, stroke, and neovascularization, commonly resulting in seizures, neurocognitive and motor delay and decline (Scott & Smith, 2009). Currently, the only available treatment is revascularization surgery to promote stable collateral blood flow and bypass stenotic vessels (Kuroda & Houkin, 2008).

The genetics of MMD are incompletely understood. Moyamoya physiology can occur as a consequence of several genetic syndromes including sickle cell disease, Alagille Syndrome, rasopathy and neurofibromatosis, so called moyamoya syndrome (Gatti, Torriente, & Sun, 2020); however, can also occur in the absence of underlying syndrome, albeit by a different mechanism. The complex environmental and genetic factors that confer risk for steno‐occlusive arteriopathy are poorly understood (Miyatake et al., 2012; Research Committee on the Pathology and Treatment of Spontaneous Occlusion of the Circle of Willis,, & Health Labour Sciences Research Grant for Research on Measures for Infractable Diseases, 2012). Genome‐wide association studies performed in East Asian populations, where the incidence of MMD is particularly high, have demonstrated an association between common variants in ring finger protein 213 (*RNF213*) and MMD development (Kamada et al., 2011; Liu et al., 2011). In particular, the p.Arg4810Lys *RNF213* variant (c.14429G > A, rs112735431) is strongly associated with MMD in East Asian populations, conferring a >100‐fold risk of disease, though with reduced penetrance, estimated at 1/150(Guey et al., 2017; Kamada et al., 2011; Liu et al., 2011). The association between *RNF213* variants has been replicated in diverse ethnic cohorts, albeit with different variants (Cecchi et al., 2014; Guey et al., 2017). Furthermore, *RNF213* gene variants have been identified in cases of familial stroke with autosomal dominant inheritance (Guey et al., 2017; Miyatake et al., 2012; Zhang et al., 2019).

*RNF213* encodes mysterin, a protein of unknown function, but with suspected E3 ubiquitin ligase and motor activity given its possession of a RING domain and two AAA+ ATPase domains (Morito et al., 2014). Though nearly ubiquitously expressed, *RNF213* deficiency studies in murine and zebrafish models suggest a prominent role in angiogenesis and blood vessel response to injury and hypoxia, with the extra‐vascular function of *RNF213* incompletely characterized (Ito et al., 2015; Lin et al., 2020; Liu et al., 2011; Sonobe et al., 2014; Wen et al., 2016).

Here, we present two cases of congenital liver, kidney, and skin disease in children with severe MMD, both found by exome sequencing to have de novo missense variants in *RNF213*. These cases suggest an *RNF213*‐spectrum syndrome defined by kidney, skin, and liver pathology in addition to MMD.

## 2 METHODS

### 2.1 Editorial policies and ethical considerations

Consent for participation was obtained from each family, and appropriate consent forms were signed. For the patient evaluated at The Children's Hospital of Philadelphia, the study was approved by the IRB (Protocol number 16‐013278).

### 2.2 Genetic testing methodology

Clinical trio exome sequencing was performed on Patient 1 at Invitae, and at Gene Dx for Patient 2. Exome at Invitae was performed by isolating genomic DNA, enriching targeted regions using a hybridization‐based protocol, and sequencing using an Illumina platform. Targeted regions (95% of mappable exome ±10 base pairs of flanking region) were covered at >×20 depth. Reads were aligned to the reference sequence (GRCh37). All reported pathogenic and likely pathogenic variants were independently confirmed either by sanger sequencing, Pacific Biosciences SMRT sequencing, or MLPA‐seq. Regarding Patient 2, after negative clinical exome testing, Patient 2 was recruited into the Center for Applied Genomics (CAG) at CHOP for exome reanalysis. Variant call format (VCF) were obtained from Gene Dx and reanalyzed using an in‐house variant annotation, filtration and prioritization platform developed within the CAG and validated for clinical use. Variants were initially filtered at 0.5% gnomAD MAF and annotated with a combination of multiple tools and databases, including Variant Effect Predictor, HGMD, ClinVar, dbSNP, OMIM, HPO, PolyPhen‐2, and SIFT, and a custom‐built splice‐site annotator. Variants are assigned a priority score of likelihood as the causal variant for the patient's disease, ranked using a weighted combination of (a) overlap with HPO terms, (b) patient and family genotypes, (c) predicted functional impact, (d) inheritance modeling, (e) presence in mutation databases such as HGMD and ClinVar; and other factors. The identified *RNF213* variant was validated by sanger‐sequencing using the following primers: F: CCTGTAAACCTAGCCCCTCAT; R:TCCCCAAGATCATGTACTAGCT. Gene Matcher was used to match these two cases (Sobreira, Schiettecatte, Valle, & Hamosh, 2015).

### 2.3 Case presentations

#### 2.3.1 Patient 1

**FIGURE 1:**
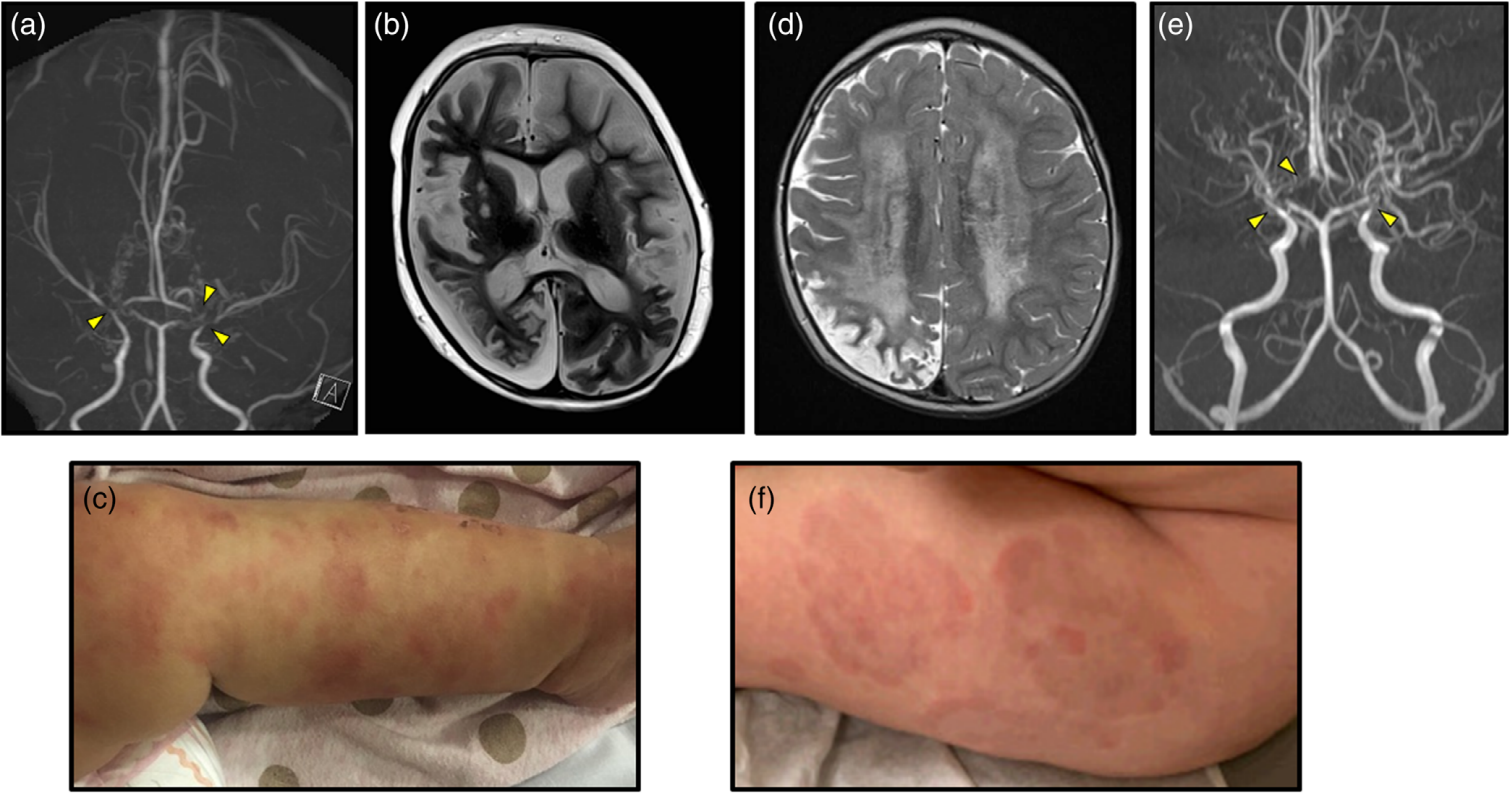
(a) Patient 1 time‐of‐flight MR angiogram technique with 3D reconstructions at 14 months showing marked narrowing of the distal ICA, right middle cerebral artery, bilateral posterior communicating arteries and anterior communicating arteries, and numerous collaterals. (b) Patient 1 T2‐weighted axial MRI at 17 months showing extensive cerebral atrophy and encephalomalacia secondary to recurrent infarction and chronic ischaemia. (c) Skin findings in Patient 1 showing multiple, flat skin lesions, some with central clearing. (d) T2‐weighted axial MRI for Patient 2 at 20 months of age showing bilateral cerebral white matter signal alteration most consistent with ischemic change, and chronic right posterior division middle cerebral artery stroke with marked encephalomalacia and gliosis, consistent with the hypoperfusion state present in a high grade moyamoya (e) Patient 2 time‐of‐flight MR angiogram technique with 3D reconstructions at 20 months showing marked irregularity and minimal to no flow related enhancement within the distal supraclinoid internal carotid arteries, A1 and M1 segments bilaterally, diminished flow related enhancement within the M2 segments and beyond, worse on the right than the left, with marked increase in bilateral lenticulostriate collaterals. (f) Skin findings in Patient 2 showing multiple, flat annular rashes with central clearing [Color figure can be viewed at wileyonlinelibrary.com]

Patient 1 was the first child of a New Zealand Maori couple, born at full‐term, following an uncomplicated pregnancy and delivery. At 4‐months of age, she presented with irritability, lethargy, and decreased left arm movement, followed by seizures. Brain MRI/MRA demonstrated right hemispheric infarction, diffuse, bilateral vasculopathy, and severe bilateral stenosis of the terminal internal carotid arteries (ICAs), proximal middle cerebral arteries (MCAs), and origin of the anterior cerebral arteries (ACAs), consistent with a diagnosis of MMD. At 9‐months of age she developed recurrent seizures and lethargy in the context of an intercurrent illness. MRI/MRA imaging showed a sub‐acute left MCA territory infarct. Vascular imaging showed progression of the proximal MCA narrowing bilaterally. At 14‐months of age, increasing lethargy and irritability prompted further MRI/MRA imaging, which identified a right posterior cerebral artery territory infarction, and new severe narrowing of the P2 segments of the posterior communicating arteries (PCAs) bilaterally. Numerous collateral vessels were present. (Figure 1a). Aspirin was administered from diagnosis. Surgical revascularization was precluded by steroid therapy and comorbidities. Family opted for palliative management, and at 17‐months of age further infarction led to her ultimate death (Figure 1b).

Skin disease was evident from the first weeks of life, initially as urticarial‐like ring skin lesions over the face and limbs, which evolved into annular, bruise‐like, and fixed plaques (Figure 1c). Skin biopsy was nonspecific, showing inflammatory melanin deposition in the upper dermis, but no features of vasculitis or other inflammation.

Elevated aminotransferases were detected within the first weeks of life (400 s U/L). These continued to rise, ultimately peaking at 7‐months of age (5000 s U/L), and then decreased without intervention. Liver echotexture was mildly coarse and bright on ultrasound. Liver biopsy at 7‐months of age showed preserved liver architecture with scattered hemosiderin‐laden histiocytes, mild portal tract expansion, and fine portal to portal tract bands of fibrosis without evidence of autoimmune hepatitis.

Patient was noted to have hematuria and nephrotic range proteinuria with normal creatinine during her initial workup. She developed hypertension, requiring multiple antihypertensive medications. Echocardiogram showed mild to moderate left ventricular hypertrophy with normal function, consistent with longstanding hypertension. Renal ultrasound was normal; CT angiogram showed bilateral renal artery stenosis. Renal biopsy performed at 9‐months of age showed numerous immature glomeruli with under‐developed capillary loops and prominent glomerular podocytes, scattered glomerulosclerosis, interstitial fibrosis and tubular atrophy. Electron microscopy findings were not suggestive of immune complex deposition. The patient maintained normal kidney function.

Ophthalmology examination was unremarkable with no evidence of retinal vasculitis or posterior embryotoxon. Autoimmune work up was negative. ESR remained normal. CSF was collected on two occasions with no evidence of CNS inflammation. White cell count and CSF neopterin were normal. The patient was initially treated with methylprednisone with improvement in skin rash, and was maintained on prednisolone. Skin rash later recurred and did not resolve with trials of etanercept, toculizumab or ruxolitinib therapy.

Trio whole exome sequencing identified two variants in *RNF213*: a de novo likely pathogenic variant in exon 62 (c.14566 T > C; p.[C4856R]), which is absent from population databases, and a paternally‐inherited missense variant of uncertain significance (c.13589C > T, p.[Ala4503Val]), with an ExAC frequency 0.02%. Father has no history of stroke, skin, liver, or kidney disease. Other identified de novo and rare variants were felt to be unrelated to phenotype (Table S1). Parents have since had a second child who had a normal brain MRI at 6‐weeks of age. No genetic testing has been done.

#### 2.3.2 Patient 2

Patient 2 was the product of a full‐term, uncomplicated pregnancy to a G2P1→2 mother. Birth weight was 3.997 kg (75–90%); length was 53 cm (75%). Poor weight gain was noted shortly after birth with associated poor feeding, and he was diagnosed with gastroesophageal reflux disease. At 4 months of age, patient was hospitalized with persistent failure to thrive and was found to have elevated aminotransferases (AST 310 U/L; ALT 317 U/L) with normal liver ultrasound, hypertension with evidence of left ventricular hypertrophy, stage III chronic kidney disease, and normal‐sized but diffusely echogenic kidneys bilaterally with no evidence of vesicoureteral reflux. Metabolic workup including plasma amino acids, urine organic acids, very‐long chain fatty acids, pipecolic acid, carbohydrate deficient transferrin, and N‐glycan testing was nondiagnostic. Protease inhibitor (PI) typing was notable for IZ, consistent with a diagnosis of alpha‐1 antitrypsin deficiency (AATD). A chromosomal microarray, congenital anomalies of the kidney and urinary tract gene panel, and a cystic kidney disease gene panel were nondiagnostic. Patient was discharged home on amlodipine monotherapy.

Patient was re‐admitted at 6‐months of life with vomiting, hypertension, and new onset rash confirmed by biopsy to be erythema multiforme. Due to concern for amlodipine allergy, patient was switched to propranolol therapy with good response. He continued to have intermittent recurrence of his rash: large, flat, red lesions with central clearing that were nonpruritic and typically appeared on the arms and legs (Figure 1f). His weight gain improved, but he continued to have short stature with normal endocrine laboratories. At 11‐months of age he was admitted due to an episode of hypoglycemia, unresponsiveness, and facial droop of unclear etiology. Trio whole exome sequencing was sent and was notable for biallelic *SERPINA1* pathogenic variants (maternally‐inherited c.1096G > A; p.(E366K) pathogenic variant and a paternally‐inherited c.187C > T; p.(R63C) pathogenic variant), consistent with his known AATD deficiency diagnosis.

At 13‐months of age he was admitted for insidious onset left arm and leg weakness and twitching. MRI revealed an acute right parieto‐occipito‐temporal stroke. MRA showed severe narrowing of the bilateral distal internal carotid arteries (ICA) as well as the proximal portions of bilateral middle cerebral arteries (MCA) and anterior cerebral arteries (ACA). Follow up MRI and MRA at 20‐months of age showed significant disease progression with bilateral cerebral white matter signal alteration encompassing nearly all deep, subcortical and periventricular white matter consistent with ischemic change; a right mainly posterior division MCA chronic infarct with marked encephalomalacia and gliosis; progressive narrowing of the bilateral distal ICAs, origin of the bilateral MCAs and ACAs with near complete loss of flow related enhancement; and multiple, small, tortuous collateral vessels, consistent with a diagnosis of severe MMD (Figure 1d,e). Abdominal MRI and MRA to look for renal artery narrowing and other vascular malformations at 20‐months of age was notable for normal renal arteries with no evidence of narrowing, normal liver, and kidneys with cystic changes bilaterally classified as congenital cystic dysplasia. Patient was managed with aspirin therapy initiated at time of stroke and underwent urgent bilateral pial synangiosis surgery at 21 months of age when repeat imaging showed marked progression of arteriopathy and persistent ischemic injury, which was well tolerated without complications. Due to the combination of MMD, elevated aminotransferases, recurrent skin rashes, and kidney dysplasia with a nondiagnostic exome, patient was referred to the Center for Applied Genomics for research‐based exome reanalysis. Reanalysis was notable for a de novo variant in *RNF213* (c.12416 T > G; p.[L4139W]), which is absent from population databases and predicted pathogenic by polyphen (score 1.000). Other identified de novo and rare variants were felt to be unrelated to phenotype (Table S2).

## 3 DISCUSSION

MMD is a progressive cerebral vasculopathy and stroke syndrome, typically of nongenetic basis, but at times demonstrating autosomal dominant inheritance with variable penetrance. *RNF213* is a well‐established susceptibility gene for MMD, with the East‐Asian founder variant p.(Arg4810Lys) conferring over a 100‐fold increase in risk of disease (Kamada et al., 2011; Liu et al., 2011; Miyatake et al., 2012). *RNF213* encodes a protein of unknown function, but with suspected roles in protein turnover (Morito et al., 2014). We have identified two patients with heterozygous de novo variants in *RNF213* with severe, early‐onset moyamoya disease and stroke, but also with novel extra‐cerebral phenotypes, including chronic kidney disease, liver disease with elevated aminotransferases, and skin disease.

The specific association between *RNF213* gene variants and pediatric MMD and stroke has been described previously, both in children heterozygous or homozygous for the founder East‐Asian p.(Arg4810Lys) variant as well as in children with rare, de novo variants (Guey et al., 2017; Ito et al., 2015; Miyatake et al., 2012; Zhang et al., 2019). The observation that individuals with biallelic *RNF213* variants have more severe and earlier‐onset disease suggests that gene‐dosage plays a critical role in dictating disease pathology, and would suggest that disease‐associated heterozygous variants in pediatric stroke have a severe and deleterious effect on *RNF213* function that cannot be overcome by the wild‐type allele.

Though the exact function of *RNF213* and the mechanism by which gene variants cause disease is unknown, in vitro and in vivo models support a role for *RNF213* in angiogenesis. Specifically, abnormal *RNF213* function in vitro causes abnormal cellular proliferation and dysregulation of inflammatory and extracellular matrix gene expression (Ohkubo et al., 2015), morpholino‐mediated and TALEN‐mediated suppression of *rnf213* expression in zebrafish causes abnormal vascular sprouting (Lin et al., 2020; Liu et al., 2011; Wen et al., 2016), and murine models of *rnf213* deficiency demonstrate abnormal angiogenesis and response to vascular injury (Ito et al., 2015; Sonobe et al., 2014). The exact mechanism by which variants in *RNF213* disrupt angiogenesis and vascular injury response is unknown, but *RNF213* is known to play a role in noncanonical Wnt and calcium signaling and NF‐kB pathway activation, which are pathways known to regulate growth and inflammatory signaling (Amal et al., 2019; Scholz et al., 2016; Takeda et al., 2020).

A role for *RNF213* outside the central nervous system has been suspected, given the near‐ubiquitous expression of the gene. Additional roles uncovered for *RNF213* include fast muscle formation and neuromuscular signaling (Kotani et al., 2015), cellular response to hypoxia (Banh et al., 2016), beta‐cell function (Kobayashi et al., 2013), and energy and lipid metabolism (Banh et al., 2016;Piccolis et al., 2019; Sugihara et al., 2019). *RNF213* promotes lipid droplet stability by interfering with adipose triglyceride lipase activity, and its deficiency protects against lipotoxicity and palmitate‐induced cell death, though interestingly the common East‐Asian variants have no effect on lipid droplets *in vitro* (Piccolis et al., 2019; Sugihara et al., 2019). Extra‐cerebral manifestations associated with *RNF213* variants have also been described clinically, including an infant who presented with elevated aminotransferases found on biopsy to have lysosomal neutral fat accumulation and cytoplasmic cholesterol crystals in addition to MMD (Harel et al., 2015), a child who presented with a dysplastic right kidney in addition to MMD (Dibi, Maana, Jabourik, & Bentahila, 2017), and a young adult with polycystic kidneys as well as MMD (Pracyk & Massey, 1989). Interestingly, in all cases where genetic data was available, mutations clustered in the *RNF213* C‐terminal region, which is characteristic of isolated MMD as well as with our syndromic patients (Harel et al., 2015).

Given the precedence in the literature of other patients with MMD and liver and kidney pathology (Dibi et al., 2017; Harel et al., 2015; Pracyk & Massey, 1989), the patient reported by Harel and colleagues (Harel et al., 2015) with MMD and liver disease with a known de novo *RNF213* variant, as well as the near‐identical phenotypes of our reported patients, both with de novo *RNF213* variants, we are highly suspicious of a distinct hepatorenal phenotype associated with *RNF213* gene variants. The etiology of the syndromic presentations for these patients is unclear. Patient 2 harbors biallelic variants in *SERPINA1*, resulting in AATD, though these variants are unlikely to explain his phenotype, as PI typing IZ usually manifests in milder liver disease in childhood (Stoller, Hupertz, & Aboussouan, 2006). There is a known association between AATD and cytoplasmic staining anti‐neutrophil cytoplasmic antibodies (c‐ANCA) vasculitis, specifically granulomatosis with polyangiitis, with increased risk in those with PI Z and S (Mahr et al., 2010); however, there have been no reports or mechanistic literature to support AATD manifesting in MMD, and patient's skin biopsy results were felt to be inconsistent with those typically seen in AATD. Therefore, we concluded the Z allele may be a disease modifier and not the main cause of phenotype.

Regarding kidney phenotype, patients had disparate findings of structurally normal kidneys with biopsy evidence of glomerulosclerosis, interstitial fibrosis and tubular atrophy (Patient 1) and cystic kidney dysplasia (Patient 2). We hypothesize that both of these patients experienced disruption to the kidney microvasculature during development as a result of their *RNF213* variants, with resultant hypoxia and diminished blood flow, and this interfered with proper kidney patterning. The exact nature of their kidney disease likely reflects the exact time in development that the disruption occurred and the degree of vascular compromise.

**FIGURE 2:**
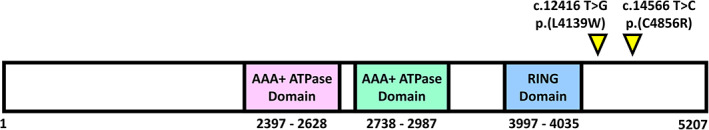
Schematic of the RNF213 protein showing the 2 AAA+ ATPAse domains as well as the RING domain. Amino acid numbers listed below the figure. Identified patient variants noted by yellow arrow [Color figure can be viewed at wileyonlinelibrary.com]

It is unknown at this time why these patients demonstrate such severe cerebral and extra‐cerebral manifestations. All variants map to the *RNF213* C‐terminal region, which has been established as a hot‐spot for severe disease (Cecchi et al., 2014; Guey et al., 2017; Zhang et al., 2019) (Figure 2). *RNF213* variants may cause disease via a threshold effect, and the variants identified in our patients may more severely impact *RNF213* function. This model is certainly supported by the finding that individuals harboring two copies of the East‐Asian *RNF213* risk allele have earlier‐onset and higher penetrant disease (Miyatake et al., 2012; Zhang et al., 2019). Patient 1 was found to harbor two changes in the *RNF213* gene, and had more severe disease with early death. Importantly, we are unable to determine whether these changes are in *cis* or *trans*. It is also possible that only certain variants affect extra‐cerebral *RNF213* function, as the East‐Asian p.(Arg4810Lys) variant is an established risk factor for MMD, but does not affect lipid droplets *in vitro* (Sugihara et al., 2019).

The skin pathology seen in our two patients has not been reported previously. Patient 1 was steroid dependent, suggesting an inflammatory component to the skin disease, whereas Patient 2 has not required treatment. Skin biopsy on Patient 1 was nonspecific but skin biopsy performed on Patient 2 was suggestive of erythema multiforme, which is an immune‐mediated condition usually triggered by viral infection or by an environmental or toxic trigger. It is tempting to speculate that the *RNF213* variants seen in these patients disrupt NF‐kB signaling and the immune response more severely than previously described variants. This phenomenon may also be relevant to the hepatitis phenotype, as hepatocytes have a TNF‐receptors and may be injured by disrupted NF‐kB signaling (He et al., 2021). Skin pathology may also be related to impaired kidney and liver function and accumulating metabolites with cutaneous toxicity; however, neither patient had a GFR sufficient low to disrupt toxin clearance and liver function was preserved in both patients.

We highlight the identical and unusual presentations of our two patients with severe liver, kidney and skin disease in addition to MMD, and suggest a novel multi‐organ syndrome associated with de novo *RNF213* gene variants. It will be interesting to determine if *RNF213* variants can produce exclusively extra‐cerebral disease with isolated kidney, liver and skin findings. Such patients would be critical in truly elucidating the biology of *RNF213* and the mechanism of disease.

## CONFLICT OF INTEREST

The authors declare no conflict of interest.

## AUTHOR CONTRIBUTIONS

Alanna Strong and Gina O'Grady helped conceptualize and design the study, evaluated the patients clinically, helped in variant interpretation and data analysis, and drafted the manuscript. Dong Li, Courtney Vaccaro, and Deborah Watson facilitated data acquisition and analysis, and contributed to manuscript preparation and review. Diana Slater and Cuiping Hou contributed to data generation. Erum A. Hartung, Evelyn Shih, Jonathan R. Bishop, Kathleen Loomes, Tamir Diamond, William Wong, Sanmati Cuddapah, and Anne Marie Cahill evaluated the patients, guided appropriate genetic evaluation, imaging studies and clinical care, and recognized each patients' atypical presentations. They also critically reviewed the manuscript. Hakon Hakonarson conceptualized and designed the study, helped in data analysis, critically reviewed and edited the manuscript, and provided funding for the work. All authors approved the final manuscript as submitted and agree to be accountable for all aspects of the work.

## Supporting information

**TABLE S1** Rare de novo and homozygous and compound heterozygous variants identified in Patient 1

**TABLE S2** Rare de novo and homozygous and compound heterozygous variants identified in Patient 2

## Data Availability

Data sharing is not applicable to this article as no new data were created or analyzed in this study.
