# Supplementary material for: A new syndrome of moyamoya disease, kidney dysplasia, aminotransferase elevation, and skin disease associated with de novo variants in RNF213
Source: Am J Med Genet A. 2021 May 7;185(7):2168–74. doi: 10.1002/ajmg.a.62215 (PMC8360119; doi:10.1002/ajmg.a.62215)
Supplement: Supplementary file 1 — TABLE S1 Rare de novo and homozygous and compound heterozygous variants identified in Patient 1 [file AJMG-185-2168-s001.docx]

**Supplemental Table One**: Rare *De Novo* And Homozygous and Compound Heterozygous Variants Identified In Patient 1
